# A Hybrid Technique Using Video Laryngoscope-assisted Flexible Bronchoscopy to Facilitate Endotracheal Intubation in Children with Anticipated Difficult Airway: A Case Series

**DOI:** 10.4274/TJAR.2024.241587

**Published:** 2025-03-21

**Authors:** K. Gunasekaran, Reesha Joshi, Pradeep Karunagaran, V.S.G. Yachendra

**Affiliations:** 1 Saveetha Institute of Medical and Technical Sciences, Department of Anaesthesiology, Tamil Nadu, India; 2 Military Medical City Hospital, Clinc of Anaesthesiology, Doha, Qatar

**Keywords:** Difficult airway, intubation, video laryngoscope, flexible bronchoscope

## Abstract

We present a case series using a hybrid technique of video laryngoscope-assisted flexible bronchoscopy to facilitate endotracheal intubation in children with anticipated difficult airway. This series describes the management of difficult airways in four paediatric cases using the hybrid technique: two cases of Apert syndrome scheduled for cranial remodelling with orbito-facial advancement, one case of an incomplete cleft palate and retrognathia scheduled for palatoplasty, and another case of Parry Romberg syndrome scheduled for a reconstructive procedure. This case series aims to highlight the value of the hybrid technique as a safe and effective intubation modality in paediatric difficult airways.

**Main Points**

• The hybrid technique of intubation is an approach combining the advantages of a video laryngoscope and a flexible bronchoscope.

• The intubation is conducted by two anaesthesiologists: one inserts the video laryngoscope into the oral cavity, improving the visual field of the airway, and the other manipulates the endotracheal tube, loaded flexible bronchoscope through the vocal cords.

• It improves the success of tracheal intubation in children with difficult airways.

## Introduction

A difficult airway can result in multiple intubation attempts, leading to airway trauma and hypoxia.^1^ Apart from direct laryngoscopy, laryngoscope blades of alternative design and size, adjuncts, video laryngoscopes (VLS), flexible intubation scopes, supraglottic airway (SGA), optical stylets, or rigid bronchoscopes could be used.^2, 3^ Flexible bronchoscope-guided intubation is considered the gold standard for paediatric difficult airway, although manipulating the device may be difficult due to a smaller airway.^4^

We report a case series of four successful endotracheal intubations in children with difficult airways using VLS as a supplement for flexible bronchoscope navigation. This manuscript adheres to the Enhancing the Quality Transparency of Health Research guidelines. Written consent was obtained from the parents or legal guardians of all patients to publish their case details.

## Case Presentations

### Case 1

An 8-month-old female infant, weighing 8 kg, with Apert syndrome was scheduled for cranial remodelling with orbitofrontal advancement. She had a cleft palate, flat occiput, midfacial hypoplasia, and orbital proptosis. Her airway examination using the Colorado Paediatric Airway Score was 12 (Table 1).^5^ Computed tomography (CT) of the neck showed an anteriorly placed larynx, near the C1 vertebra, and a small mandibular space.

Intravenous (IV) dexmedetomidine infusion at 1 µg kg^-1^ hr^-1^, glycopyrrolate at 10 µg kg^-1^, and dexamethasone at 0.1 mg kg^-1^ were given, with intermittent IV ketamine at 0.5 mg kg^-1^ boluses. Nebulization along with superior laryngeal nerve and transtracheal blocks with 2% lignocaine was administered.

**Figure 1:**
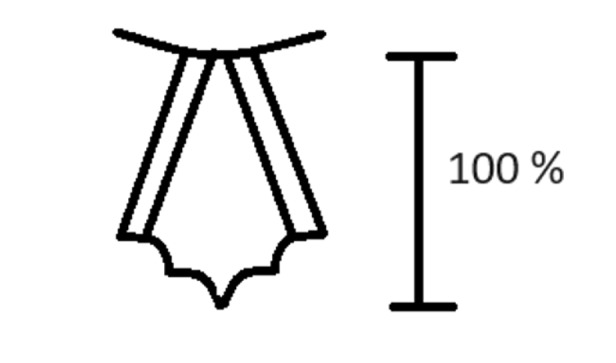
The percentage of glottic opening (POGO) score for laryngeal grading. The POGO score represents the linear span from the anterior commissure to the interartytenoid notch.

Oral intubation with the H-SteriScope, a paediatric flexible video bronchoscope (FOB) manufactured by Vathin Medical Instrument Co. Ltd, Hunan, China, with an outer diameter of 2.2 mm, was attempted alongside oxygen supplementation through nasal prongs to prevent desaturation. Owing to the narrow oral cavity, the FOB had little room, and intubation was unsuccessful. A second attempt was made with the McGrath MAC VLS (Aircraft Medical Ltd., Edinburgh, UK) with a size 1 blade. Due to the anterior larynx and inadequate angulation of the stylet or bougie, advancing the endotracheal tube (ETT) or bougie into the trachea was unsuccessful, despite a percentage of glottic opening score of 25% (Figure 1). The patient experienced a drop in saturation, and the attempt was abandoned, and the patient was ventilated.

**Figure 2:**
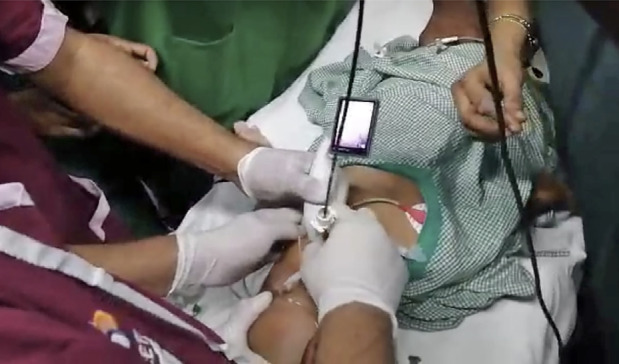
In first case with Apert syndrome a video laryngoscope is introduced into the oral cavity by one anaesthesiologist to improve visualisation of the airway and the second anaesthesiologist simultaneously manipulates the fibreoptic bronchoscope loaded with an endotracheal tube into the vocal cords.

Finally, FOB and VLS were used simultaneously by anaesthesiologists experienced in this technique. Intubation was conducted by two anaesthesiologists: one inserted the VLS into the oral cavity to improve the visual field of the airway, and the other manipulated the FOB (Figure 2). The tip of the bronchoscope, as visualised on the VLS, could be manoeuvred and passed through the vocal cords; followed by smooth insertion of the 3.5 mm ETT. After confirming adequate ventilation with capnography, the ETT was fixed.

 IV fentanyl 2 µg kg^-1^ and atracurium 0.5 mg kg^-1^ were given. Anaesthesia was maintained with dexmedetomidine at 0.5 µg kg^-1^ hr^-1^, and with isoflurane at 0.8-1.0 minimum alveolar concentration (MAC). After the procedure, the patient was shifted to the paediatric intensive care unit (PICU) and extubated the next day.

### Case 2

**Figure 3:**
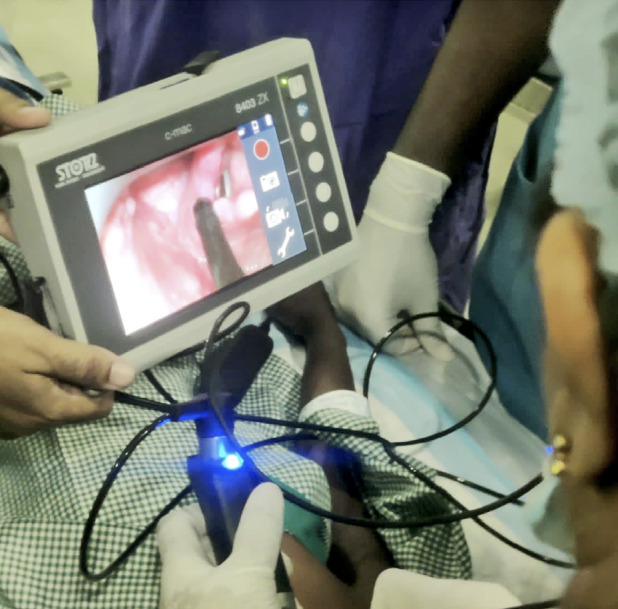
In case 2 with Parry Romberg syndrome the fibreoptic bronchoscope is manipulated into the vocal cords after visualization under the C-MAC video laryngoscope.

A 9-year-old child, weighing 19 kg, with Parry Romberg syndrome comprising right progressive hemifacial atrophy with en coup de sabre deformity, was scheduled for free anterolateral thigh adipofascial flap. Difficult intubation was anticipated again as the COPUR score was 13. CT showed hypoplasia of the right mandible. After securing IV access, IV fentanyl 2 µg kg^-1^, and propofol 2 mg kg^-1^ were administered. After confirming adequate mask ventilation, atracurium 0.5 mg kg^-1^ was administered. A C-MAC VLS (Karl Storz GmbH, Tuttlingen, Germany) with a Macintosh blade 2 was inserted. Following this H-SteriScope, a flexible video bronchoscope, with an outer diameter of 3.2 mm, loaded with 5 mm cuffed armoured ETT, was passed under VLS guidance through the vocal cords and intubation was accomplished successfully in the first attempt (Figure 3). Upon completion of the surgery, the patient was shifted to the PICU and extubated the next day.

### Case 3

A 1-year-old child, weighing 9 kg, with an incomplete cleft palate and retrognathia, was scheduled for palatoplasty. The patient had a history of difficult intubation. The COPUR score was 14. IV access was secured after inhalation induction with oxygen and sevoflurane. IV fentanyl, 2 µg kg^-1^, and atracurium 0.5 mg kg^-1^ were administered after adequate mask ventilation. A McGrath MAC VLS with a size 1 blade was inserted, and the arytenoids were visualised. An H-SteriScope, a flexible bronchoscope with an outer diameter of 2.2 mm and loaded with a 3.5 mm cuffed armoured ETT, was passed, and the child was intubated without any complications. The child was extubated at the end of surgery.

### Case 4

A 1-year-old child with Apert syndrome and craniosynostosis, weighing 7 kg, was scheduled for bilateral fronto-orbital advancement. The child had a cleft palate. The COPUR score was 13. CT neck showed an anteriorly placed larynx and a small mandibular space. After premedication with oral midazolam 0.5 mg kg^-1^, inhalation induction was performed with oxygen and sevoflurane. Following IV cannulation, fentanyl 2 µg kg^-1^ and atracurium 0.5 mg kg^-1^ were administered after adequate mask ventilation. Following visualisation of the arytenoids using a McGrath MAC VLS with a size 1 blade, an H-SteriScope, a paediatric flexible bronchoscope with an outer diameter of 2.2 mm, loaded with a 3.5 mm cuffed armoured ETT, was passed. Anaesthesia was maintained with oxygen, air, and isoflurane, (MAC 0.8-1). The child was extubated on the table.

## Discussion

Ours is a tertiary centre catering to all specialities, and our paediatric cases comprise primarily cleft repair or neurosurgical and abdominal cases. In a child with a difficult airway, alternative techniques are imperative to ensure successful intubation.^6^ We ensured the availability of various sizes of Mackintosh and Miller laryngoscope blades, adjuncts like bougies and stylets, VLS with different sized blades, flexible bronchoscopes and invasive access. SGAs were available in all cases as a backup plan except the first case, where there was a leak in the size 1.5 ProSeal on the day of the procedure.

SGAs allow effective oxygenation and ventilation by relieving upper airway obstruction as they displace the tongue and the soft tissue of the posterior pharynx. SGAs can be used as the primary device in recognized difficult airways.^7^ However, considering limited access in head and neck procedures or the duration of the case, we decided to intubate our patients.

Intubation through a SGA can be a suitable option in difficult airways by acting as a conduit for the FOB loaded with an ETT to pass through. While it restores the oxygenation and ventilation, it is important to avoid inadvertent extubation when removing the SGA, as the ETT may extend only a short distance beyond the distal tip of the SGA.^7^

VLS is associated with better glottic visualisation, higher success rate (92%), and a faster learning curve.^8^ However, despite a good glottic view, it does not always aid in the easy passage of an ETT, as was the case in our first instance, due to the misalignment of the axes between the optical visualisation of the vocal cords and ETT introduction.^8^ The failure rate of VLS is 2% as a primary technique and 8% as a rescue technique.^9^

Flexible bronchoscopy is the gold standard for elective difficult intubation.^10^ It is associated with a higher success rate of intubation compared to VLS in patients with difficult airways.^11^ On the downside, manipulating the device could be difficult due to the smaller airway in children, resulting in poor visualization.^4^ H-SteriScope is a new single-use flexible video-bronchoscope (Vathin Medical Instrument Co. Ltd, Hunan, China), designed with outer diameters of 2.2-6.2 mm. Except for the 2.2 mm bronchoscope, the other bronchoscopes have a working channel with diameters ranging from 1.2-3.2 mm.^12^

We have described a hybrid technique using a VLS to assist in flexible bronchoscopy for endotracheal intubation. The VLS is inserted by one anaesthesiologist, who improves visualisation of the glottis, while the flexible bronchoscope with a mounted ETT is inserted orally and manipulated through the vocal cords by a second anaesthesiologist. In a simulated study, it was shown that a single anaesthesiologist can introduce the laryngoscope, which can be held in place by a second person without airway training.^13^

The hybrid technique showed a greater first-attempt intubation success rate in adult patients compared to either individual technique.^13, 14^ The hybrid technique would facilitate easier and quicker intubation, thereby minimizing episodes of desaturation. In our case series, we did not observe any significant oxygen desaturation or bradycardia events. By choosing this technique, we were able to intubate the children safely in a much shorter time, average 60 seconds. Proper communication between team members is of prime importance for this technique to be effective.

Since ventilation with face mask was adequate, we paralysed a few of our cases before attempting intubation. Due to unavailability of sugammadex in this part of the world at the time of conducting the cases and in order to avoid possible bradycardia with succinylcholine, we used atracurium. When available, rocuronium and sugammadex are better alternatives to succinylcholine and atracurium in patients with difficult airways.^15^

## Conclusion

The hybrid technique of VLS-assisted flexible bronchoscopy facilitates safe and successful tracheal intubation in children with difficult airways, and can be used electively or as a rescue measure.

## Ethics

**Informed Consent:** Written consent was obtained from the parents or legal guardians of all patients to publish their case details.

## Figures and Tables

**Table 1. Colorado Paediatric Airway Score (COPUR):** 

**COPUR**		**Points**
**C: Chin** From the side view, is the chin: · Normal size? · Small, moderately hypoplastic? · Markedly recessive? · Extremely hypoplastic?		- - 1 2 3 4
**O: Opening** Interdental distance between the front teeth: · 40 mm · 20-40 mm · 10-20 mm · <10 mm		- - 1 2 3 4
**P: Previous intubations, obstructive sleep apnoea (OSA)** · Previous intubations without difficulty · No past intubations, no evidence of OSA · Previous difficult intubations, or symptoms of OSA · Difficult intubation-extreme or unsuccessful; emergency tracheotomy; unable to sleep supine		- 1 2 3 4
**U: Uvula Mouth open, tongue out, observe palate** · Tip of uvula visible · Uvula partially visible · Uvula concealed, soft palate visible · Soft palate not visible at all		- 1 2 3 4
**R: Range** Observe line from ear to orbit, estimate range of movement, looking up and down · >120**°** · 60**°**-120**°** · 30**°**-60**°** · <30<strong>°</strong>		- - 1 2 3 4
**Modifiers: add point for** · Prominent front “buck” teeth · Very large tongue, macroglossia · Extreme obesity · Mucopolysaccharidoses		- 1 1 1 2
**Prediction points**	**Intubation difficulty**	**Glottic view**
5-7	Easy, normal intubations	1
8-10	More difficult, laryngeal pressure may help	2
12	Difficult intubation, fibreoptic less traumatic	3
14	Difficult intubation, requires fibreoptic or other advanced methods	3
16	Dangerous airway, consider awake intubation, advanced methods, potential tracheotomy (Patients with hypercarbia	4
16+ scores	> 16 are usually incompatible with life without an artificial airway	-
